# Effects of Viewing Cute Pictures on Quiet Eye Duration and Fine Motor Task Performance

**DOI:** 10.3389/fpsyg.2020.01565

**Published:** 2020-07-10

**Authors:** Naoki Yoshikawa, Hiroshi Nittono, Hiroaki Masaki

**Affiliations:** ^1^Faculty of Sport Sciences, Waseda University, Saitama, Japan; ^2^Graduate School of Human Sciences, Osaka University, Osaka, Japan

**Keywords:** cute pictures, kawaii, quiet eye, choking under pressure, fine motor skill

## Abstract

We investigated if viewing cute pictures could improve fine motor skills and prolong quiet eye (QE) duration. QE is a gaze phenomenon, and its duration (i.e., the period between fixation onset preceding a critical movement and fixation offset) is thought to represent attention control. As it has been reported that QE duration is longer for expert athletes than for novice athletes in various sports and becomes shorter even for experts who choke under pressure during games, resulting in performance deterioration, QE prolongation is important to prevent choking under pressure. Separately, several studies have confirmed that viewing cute pictures can induce focal attention, thus improving performance in fine motor tasks. We hypothesized that viewing cute pictures may modulate attention control and prolong QE duration. We also tested if the beneficial effects of viewing cute pictures could be obtained in a high-pressure situation in which participant performance was evaluated by an experimenter. We used a fine-motor task requiring participants to use a pair of tweezers to remove 12 small pieces from holes in a game board. We randomly assigned participants to either the baby-animal pictures group or the adult-animal pictures group, based on pictures viewed prior to the task. Participants executed the task in a pre-test, post-test, and pressure test. In both the post-test and the pressure test, participants viewed seven photographs of either baby animals or adult animals before execution of the task. In accordance with previous research, task precision increased after viewing pictures of baby animals in both the post-test and pressure test. Furthermore, QE duration was also prolonged after viewing cute pictures in the post-test, but not in the pressure test. Neither performance improvement nor QE prolongation was found after viewing pictures of adult animals. These results suggested that simply viewing cute pictures could prolong QE duration without pressure and might provide a beneficial effect on performance, even in a high-pressure situation.

## Introduction

Research has shown that viewing cute pictures (e.g., puppies and kittens) may narrow the focus of attention and increase the preciseness of fine motor skills, whereas these performance improvements may not occur after viewing pictures of adult dogs and cats (Sherman et al., 2009; Nittono et al., 2012). Nittono et al. (2012) carefully conceptualized “kawaii,” a Japanese word that roughly corresponds to “cute” as an emotion induced by perceiving cuteness, and asserted that kawaii has an inherent beneficial effect on fine motor skills that require focal attention. The beneficial effect of induced focal attention was also found in other tasks that did not depend on motor skills. In a visual search task, participants’ correct responses increased within a time limit following observation of cute pictures, and in a global–local letter task, participants attended to local aspects more than global aspects of the stimuli, resulting in narrower attentional focus (Nittono et al., 2012).

According to Nittono (2016), kawaii is a positive affect that may be mediated by baby schema features composed of roundness of the face, forehead-to-face ratio, location of the eyes, and shortness of both the hands and legs (Lorenz, 1943). Kawaii is associated with moderate arousal, approach motivation (i.e., wanting to get closer), and sociality, but not associated with threat or withdrawal (Nittono, 2016). Glocker et al. (2009) reported that possible neural correlates of baby schema were the right nucleus accumbens, left anterior cingulate cortex, left precuneus, and left fusiform gyrus (however, see Bos et al., 2018 for null results in the same task). Among these brain regions, the precuneus is known to be associated with attention control (e.g., Le et al., 1998).

The link between kawaii feelings and attention is twofold. First, cute stimuli such as infant faces attract attention via bottom-up processes (Brosch et al., 2007; Lucion et al., 2017). This process can occur at a very early stage of visual processing (Brosch et al., 2008) and lasts for several seconds (Nittono and Ihara, 2017). Second, viewing cute pictures has an aftereffect on subsequent task performance, as described at the beginning of this paper (Sherman et al., 2009; Nittono et al., 2012). The latter effect should be based on top-down attentional control, because cute pictures are out of sight during task execution. There are three possible accounts for this beneficial aftereffect. First, viewing cute pictures would compel people to be more physically tender in their motor behavior and would further increase their performance (Sherman et al., 2009). Second, it would induce a strong approach motivation that has been shown to narrow attentional focus and, thus, improve task performance (Gable and Harmon-Jones, 2008; Noguchi and Tomoike, 2016). Third, viewing cute pictures would motivate caregiving behavior, which increases systematic processing of the environment with close attention to the target’s needs (Griskevicius et al., 2010). These accounts are not mutually exclusive and explain the effects of viewing cute pictures.

As this new approach for inducing focal attention has only been investigated in the last few years (Sherman et al., 2009, 2013; Nittono et al., 2012; Kringelbach et al., 2016; Karreman and Riem, 2019), more studies are needed to clarify the effects of viewing cute pictures. For example, it is unknown whether the beneficial effects can be observed in high-pressure situations. As discussed below, the utility of viewing cute pictures would be magnified if its beneficial effects are revealed for more dexterous motor skills during sporting events, because many athletes fail under severe pressure. More empirical evidence associated with pressure manipulation would be needed to apply the kawaii-viewing effects to the field of sports. Thus, we designed the present study to test if viewing cute pictures can increase preciseness of fine motor skills under pressure. If we could obtain beneficial effects in the pressure condition, this new approach may provide significant implications for preventing failure in sports as well as social communication.

Not only novice but also expert athletes may experience “choking under pressure” when playing sports. Choking under pressure has been defined as deterioration of performance despite making efforts to achieve success (e.g., Baumeister, 1984), and is often induced by competitive anxiety (Jones, 1995). Reacting emotionally to a stressful situation, an athlete is apt to become confused and lose concentration (Jones, 1995). Thus, choking under pressure is a manifestation of complex negative emotions.

Researchers have investigated the underlying mechanism of choking under pressure (e.g., Eysenck, 1979; Masters, 1992), proposing two contradictory views of the phenomenon. Convincing accounts, including the conscious processing hypothesis (Masters, 1992), self-focus model (Baumeister, 1984), and explicit monitoring theory (Beilock and Carr, 2001), assert that choking under pressure may occur when automaticity of actions is broken in a high-pressure situation (e.g., existence of audience) and shifts to controlled processing (i.e., requiring more attention). Thus, inward attention becomes a hindrance to performing well-learned motor skills. Conversely, the distraction theory (Wine, 1971) emphasizes the importance of attentional resources that should be allocated to the primary task. When other irrelevant aspects (e.g., evaluation by an audience) deprive athletes of attentional resources for their primary task, performance deterioration may occur. It is therefore plausible that attention could either harm or contribute to performance achievement, depending on an athlete’s skill level.

Although the underlying mechanism of choking under pressure has been studied, new effective methods for its prevention that can be carried out easily in any pressure situation need to be developed. Traditionally, relaxation techniques (e.g., autogenic training; Schultz and Luthe, 1959) have been applied to prevent choking under pressure (Lehrer, 1996). However, relatively long-lasting training is required to acquire a relaxation technique under the supervision of an expert. Therefore, athletes are apt to drop out from the effortful training.

Among prevention techniques for choking, sport psychologists have recognized benefits of quiet eye (QE) training (Harle and Vickers, 2001), in which athletes consciously prolong their gaze duration on an external target toward which they make critical actions. QE is a gaze behavior associated with a final fixation directed toward a specific location or object, within one or three degrees of visual angle for a minimum of 100 ms, that occurs before the onset of a critical movement (Vickers, 2007; Causer et al., 2014a, b). It has been reported that QE duration is shorter for novice than expert athletes (Vickers, 1996; Janelle et al., 2000; Williams et al., 2002; Gonzalez et al., 2017a), longer for successful trials, as opposed to unsuccessful trials (Wilson and Pearcy, 2009), and shorter when a skilled athlete experiences choking under pressure during a game (Behan and Wilson, 2008; Wilson et al., 2009).

Based on these findings, a new prevention technique, referred to as “QE training,” has been developed. After verbal-instruction-based training conducted for several weeks, athletes learned to prolong their QE duration, and later succeed in preventing choking under pressure during actual games (Vine and Wilson, 2010, 2011). QE training can be extended to improve different types of complex motor skills, including surgical knot-tying (Causer et al., 2014a, b). The most advantageous aspect of QE training is its simplicity and effectiveness, compared to traditional relaxation techniques.

However, the reason why prolonged QE likely yields better performance remains unclear. So far, several possible explanations have been proposed. According to a comprehensive review of QE phenomena (Gonzalez et al., 2017b), the primary causal mechanisms of QE can be attributed to several processes, including motor programming, attention, inhibition, and online control. Except for motor programming, the other processes are mediated by attention control. In general, a longer QE duration may represent attention allocated to relevant objects, suppressing processes of irrelevant stimuli (Vickers, 1992, 1996; Klostermann et al., 2014).

For the motor programming account, QE duration is thought to represent the period during which neural networks are organized to control an elaborate movement (Vickers, 1996; Williams et al., 2002; Behan and Wilson, 2008). Williams et al. (2002) recorded gaze behaviors of highly skilled and novice billiard players, manipulating the complexity of billiard shots. They found that longer QE duration was associated with shot difficulty, players’ skill levels, and better performance, suggesting that QE duration represents a critical period for proper cognitive processing. Recording Bereitschaftspotential (BP), a previous study found long-lasting BPs during longer QEs, indicating deliberate motor programming (Mann et al., 2011).

Other studies also suggested that QE training might be a useful intervention to enhance attention control and maintain performance under high-pressure conditions, eliminating adverse effects of anxiety on attention control (Behan and Wilson, 2008; Vine and Wilson, 2010, 2011; Vine et al., 2011). Thus, it is plausible that both attention control and motor programming are involved in preventing performance deterioration.

Although accumulated evidence provides support for QE training as one of the most feasible techniques for preventing choking under pressure, an eye-tracker system is a necessary tool to evaluate whether a trainee indeed learns to prolong QE duration. This might be a technical limitation of QE training. Although a recent study suggested that electrooculogram recording could evaluate QE as a replacement for an eye-tracker system (Mann et al., 2011; Gonzalez et al., 2017a; Gallicchio et al., 2018; Gallicchio and Ring, 2019), this still requires equipment for psychophysiological recordings. Therefore, it could be beneficial if a reliable method that guarantees prolongation of QE without any special equipment is established.

Given that viewing cute pictures expedites focal attention, it is reasonable to presume that QE duration would also be prolonged after viewing cute pictures. Viewing cute pictures enhances processes associated with capturing attention (Brosch et al., 2007, 2008), motivation for approach (Nittono et al., 2012), and tender and careful behavior (Sherman et al., 2009). Although both QE and kawaii-viewing effects are involved in top-down attention control, beneficial effects of viewing cute pictures rely on the extent to which induced focal attention can persist during the task, whereas QE behavior toward a specific location occurs during a specific phase of the given task. Nevertheless, it would be important to clarify that viewing cute pictures can prolong QE durations even in a pressure situation to expand the utility of the kawaii effects. Vine et al. (2011) reported that goal-oriented attentional system was deteriorated by a pressure manipulation for individuals who did not receive a QE training. This suggests that pressure manipulation is a necessary procedure to clarify the relationship between QE and top-down attention control. Because we are keen to test the kawaii effect that may facilitate top-down attention control, pressure manipulation would be helpful in revealing characteristics of the kawaii effect in association with top-down attention control. To the best of our knowledge, there is no study that tested kawaii-viewing effects under pressure.

In this study, we tested the hypothesis that viewing cute pictures may not only induce focal attention but also prolong QE duration. To do so, we used a fine motor task similar to Sherman et al. (2009) and Nittono et al. (2012), in which participants were required to use tweezers to pick up small objects out of individual holes in a game board, without touching the holes’ edges. Additionally, we tested whether these effects would also exist under pressure wherein participants were anxious about being evaluated.

Regarding attention control, previous studies have examined the effects of external and internal attention on performance. Generally, it has been concluded that attending to body parts (internal attention) results in worse performance, whereas attending to external objects, such as goal or tools (external attention), yields better performance (Wulf, 2013). For example, players showed better performance when attending to a golf club rather than their arms in a putting task (Wulf et al., 1999; Wulf and Su, 2007), when attending to a ball rather than their legs in a lofted soccer-pass task (Wulf et al., 2002), and when attending to a dartboard rather than their throwing arm in a darts game (Marchant et al., 2007, 2009). These findings imply that external attention control might be crucial for preventing choking under pressure. Although there is a report that states that prolongation of QE may not be facilitated by external attentional control (Klostermann et al., 2014), gaze at a specific location itself (e.g., a specific point on the ball immediately before a backswing of golf putting that may result in a success, Vickers, 2007) represents control over external attention.

As previous studies that reported beneficial effects of viewing cute pictures did not manipulate pressure, it is worth testing if viewing cute pictures can improve performance, even in a high-pressure situation. Pressure is a factor, or combination of factors, that may increase the importance of performance in certain situations (Baumeister, 1984). Numerous studies of athletes manipulated pressure by creating a situation where an audience evaluated performance (e.g., Beckmann et al., 2013; Gröpel and Beckmann, 2017). Hence, we conducted a pressure test in which an examiner sat beside participants and conducted an on-site evaluation of their performance during participants’ execution of a fine motor task. If a longer QE duration could be observed, even under pressure, viewing cute pictures before execution of a critical movement would be one of the most efficient ways to prevent choking under pressure. If QE duration is not found to be influenced by viewing cute pictures, regardless of performance improvement, it may be concluded that prolongation of QE would require relatively longer training, in accordance with previous findings.

## Materials and Methods

### Participants

Twenty-eight university students (mean age 20.71, SD = 1.21) participated in this study. They were assigned to either the baby-animal pictures group (eight men and seven women) or the adult-animal pictures group (five men and eight women). One participant in the baby-animal pictures group was excluded from analysis due to a technical problem with recording gaze data. All participants were right-handed with a mean handedness score of +82 on the Edinburgh Handedness Inventory (Oldfield, 1971). Written informed consent was obtained from all participants. After the experiment, participants were debriefed regarding the study’s purpose. This study was approved by the Waseda University Ethics Committee.

### Task

We used a game for children (Bilibili Dentist game, Megahouse, Tokyo, Japan) as a fine motor task, similar to tasks used by Sherman et al. (2009) and Nittono et al. (2012). In this task, using a pair of tweezers, participants removed 12 small pieces (i.e., viscera parts) from holes in a patient’s body that was drawn on a game board. Both dropping a piece while removing it and touching the edge of a hole with tweezers were classified as a failure. After a demonstration of the task by the experimenter, each participant performed the task at his/her own pace and tried to achieve the highest score among participants. The holes were sequentially numbered, indicating the order of trials. Regardless of success or failure for each hole, the participant was asked to perform the next hole immediately and complete all 12 trials. Both percentage of successful attempts and time taken to complete the task were recorded as performance outcomes.

### Procedure

Before the experiment, participants were equipped with eye tracker goggles, and calibration was conducted. During the experiment, participants placed their chins on a rubber band to fix their face position and keep the distance constant between the board and eyes (i.e., about 40 cm). We conducted three test blocks for each group, in the following order: pre-test, post-test, and pressure test. Immediately before the post- and pressure tests, participants were given seven sheets of paper (210 mm × 297 mm), each of which had a printed color photograph of an animal and asked to rank the images according to personal preference in 1.5 min. Seven images including puppies and kittens were used for the baby-animal condition, and seven images including dogs, cats, and a lion were used for the adult-animal condition. These royalty-free images downloaded from the Internet were selected according to a pilot survey, in which images of baby and adult animals differed in subjective rating scores on cuteness, infantility, and wanting to get closer, but did not differ in scores on pleasantness and excitement, in accordance with a previous study (Nittono et al., 2012). The same set of pictures was used for both the post-test and pressure test. After the pressure test, participants were asked to view the seven pictures and rate them on six-point scales in terms of cuteness, infantility, pleasantness, excitement, and wanting to get closer, ranging from 1 (not at all) to 6 (very much).

### Pressure Manipulation

To create a high-pressure situation, several ego stressors were used. In the pressure test, performance was evaluated on site by an additional experimenter, who appeared unexpectedly and sat beside the participant. Additionally, a video camera was positioned in front of participants to record their performance. Participants were told that their performance would also be evaluated offline by experimenters and a psychology expert.

Furthermore, the following false instructions were given to participants: (a) “results in the pressure test will be revealed and shared with all participants later”; (b) “you will receive either a monetary reward of 50 yen multiplied by the number of successes that exceed the mean score among all participants, or a monetary loss of 50 yen multiplied by the number of failures that fall below the mean score”; (c) “you have to continue to perform the task until you exceed the reference score calculated according to previous findings”; and (d) “if your score is extremely poor relative to the mean score among participants in the pressure test, you should participate in an additional experiment to receive honorarium.” These instructions were carefully dissolved in debriefing after the experiment by explaining the aim of the procedure.

### Recordings and Data Analysis

#### Behavioral Data

We scored both the percentage of participants’ successful attempts (i.e., “percent success”) and time taken to complete the task. To calculate percent success, the number of successful trials was divided by the total number of pieces to be picked up (i.e., 12) and multiplied by 100. Time taken to complete the task was calculated in seconds as the period between the task’s starting cue to when participants removed the tweezers from the 12th piece.

#### State Anxiety

Cognitive state anxiety was measured using form Y-1 of the State-Trait Anxiety Inventory (STAI, Spielberger and Gorsuch, 1983). The STAI form Y-1 is a 20-item self-report measure that assesses how respondents feel in the moment, in relation to each item. Participants were asked to rate each item on a four-point Likert scale, ranging from 1 (not at all) to 4 (very much so). The total score for all 20 items was used for analysis. In this study, the Japanese version, the STAI-JYZ (Hidano et al., 2000), was used because it is regarded to better assess Japanese cultural factors. Participants completed the STAI-JYZ form Y-1 before viewing pictures both in the post-test and pressure test.

#### QE Duration

Gaze behavior was recorded using an eye-tracker system (Tobii Technology Inc., Tobii Pro Glasses2, 30 Hz). We measured QE duration in milliseconds using the Tobii Glasses Analysis Software (Tobii Technology, Inc.). In this study, QE was defined as the final fixation or tracking gaze located on each target piece to be picked up, within a 1° visual angle, for a minimum of 100 ms, according to Causer et al. (2014a, b). We identified QE initiation (i.e., the QE onset) before the tweezer grips grasped a piece. Thus, QE duration represented the interval between the onset of QE (lasting more than 100 ms) and the offset of QE that occurred when the gaze deviated from the object or location by more than 1° of visual angle for a minimum of 100 ms. Therefore, QE started during the approaching movement with the tweezer and QE was terminated either immediately before or after the grasping movement. We defined the time point of grasping the piece as the final critical movement. However, determination of the final critical movement did not affect QE measures, because QE duration represented the interval between the gaze onset and the gaze offset.

We scored QE duration in each trial, although task difficulty (and thus time taken to complete a trial) might have differed among 12 trials, due to target pieces having different shapes. Both median and mean QE duration were scored considering variabilities of time taken to complete a trial. Additionally, we analyzed the proportion of QE duration to completion time in a test (i.e., total QE duration during execution of 12 pick-ups). We divided total QE durations through 12 pick-ups by the total operation time through 12 trials.

#### Statistical Analysis

Dependent variables of accuracy (i.e., percent success), time taken to complete the task, state anxiety scores, QE duration, and proportion QE duration were subjected to a two-factor mixed design analysis of variance (ANOVA) with group (baby/adult animals) as a between-subjects factor and test (pre/post/pressure) as a within-subjects factor. We also compared the mean QE duration between failed and successful pick-ups. QE duration was subjected to a three-factor mixed design ANOVA with group (baby/adult animals) as a between-subjects factor and test (pre/post/pressure) and trial result (success/failure) as a within-subjects factor. Furthermore, we analyzed operation times in both the post-test and the pressure test, using a two-way (group: 2 × test: 2) analysis of covariance (ANCOVA) with mean operation time in the pre-test as a covariate.

When a significant main effect or interaction was obtained, *post hoc* mean comparisons were performed using the Bonferroni correction. Where the sphericity assumption was violated, the Huynh-Feldt correction was applied. An independent *t*-test was performed on the subjective ratings data (baby/adult pictures). Effect sizes were calculated using partial eta squared (η_p_^2^) for omnibus comparisons and Cohen’s *d* for simple comparisons. Significance was set at *p* < 0.05 for all tests. Statistical analysis was conducted using SPSS for Macintosh, Version 25.0 (IBM Corp., Armonk, NY, United States).

## Results

### Subjective Ratings

Table 1 shows subjective ratings on each emotion item in both groups. Scores were significantly higher in the baby-animal pictures group than in the adult-animal pictures group on cuteness [*t*(26) = 2.43, *p* = 0.022, *d* = 0.92], infantility [*t*(26) = 6.66, *p* < 0.001, *d* = 2.53], and wanting to get closer [*t*(26) = 2.08, *p* = 0.047, *d* = 0.79]. Score on pleasantness tended to be higher in the baby-animal pictures group [*t*(26) = 1.86, *p* = 0.075, *d* = 0.70], although no difference was found between groups for excitement [*t*(26) = 1.49, *p* = 0.150, *d* = 0.57].

**TABLE 1:** Mean subjective rating scores on the photo images (standard error of mean in parentheses).

	Baby animals	Adult animals
Cute	4.70 (0.19)	3.88 (0.28)
Infantile	4.51 (0.20)	2.81 (0.15)
Pleasant	4.20 (0.19)	3.66 (0.22)
Exciting	2.80 (0.21)	2.38 (0.17)
Wanting to get closer	4.52 (0.25)	3.68 (0.33)

### Percent Success

**FIGURE 1:**
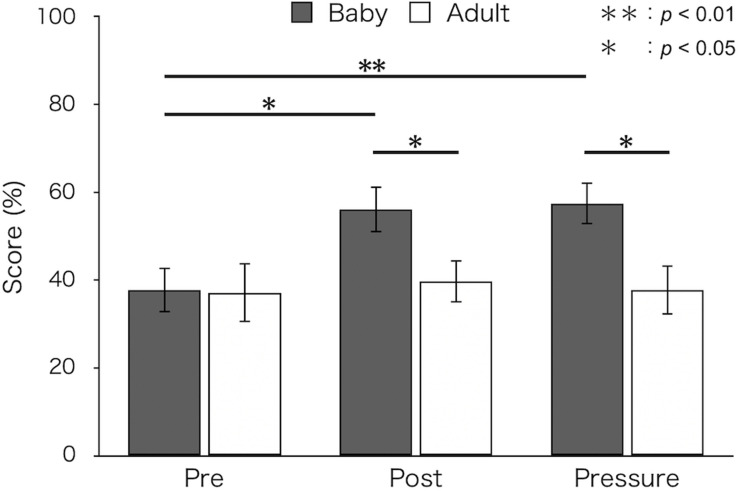
Mean percent success of the two groups in each test (Baby: viewing pictures of baby animals; Adult: viewing pictures of adult animals). Error bars indicate standard error of the mean (SEM).

Figure 1 shows mean percent success for both groups. A two-way ANOVA revealed a significant main effect of test [*F*(2,52) = 4.66, *p* = 0.018, ε = 0.88, η_p_^2^ = 0.15]. The interaction between group and test was also significant [*F*(2,52) = 3.41, *p* = 0.047, ε = 0.88, η_p_^2^ = 0.12]. A simple main effect of test was found in the baby-animal pictures group [*F*(2,52) = 8.57, *p* = 0.001]. Bonferroni-adjusted *post hoc* tests showed that percent success was significantly higher in the post-test (*M* ± *SEM* = 56.11 ± 5.24%; *p* < 0.05) and the pressure test (*M* ± *SEM* = 57.47 ± 4.82%; *p* < 0.01) than in the pre-test (*M* ± *SEM* = 37.78 ± 5.13%) for the baby-animal pictures group. Percent success was higher for the baby-animal pictures group than for the adult-animal pictures group in the post-test [baby: *M* ± *SEM* = 56.11 ± 5.24%; adult: *M* ± *SEM* = 39.74 ± 4.83%; *F*(1,26) = 5.16, *p* = 0.032] and the pressure test [baby: *M* ± *SEM* = 57.47 ± 4.82%; adult: *M* ± *SEM* = 37.82 ± 5.63%; *F*(1,26) = 7.12, *p* = 0.013]. There were no differences between groups in the pre-test [baby: *M* ± *SEM* = 37.78 ± 5.13%; adult: *M* ± *SEM* = 37.18 ± 6.71%; *F*(1,26) = 0.01, *p* = 0.943].

### Time Taken to Complete the Task

**FIGURE 2:**
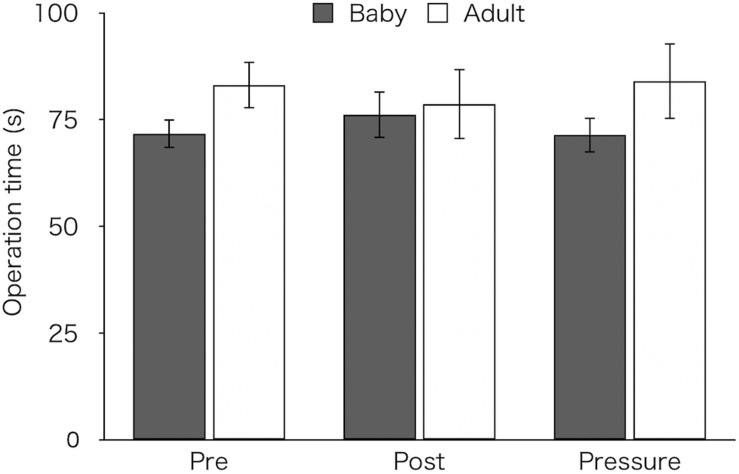
Mean time to complete task of the two groups (Baby vs. Adult) in each test. Error bars indicate standard error of the mean (SEM).

Figure 2 shows mean time taken to complete the task in both groups. A two-way ANOVA revealed neither a significant main effect [Group: *F*(1,26) = 1.28, *p* = 0.269, η_p_^2^ = 0.05, Test: *F*(2,52) = 0.01, *p* = 0.981, ε = 0.80, η_p_^2^ < 0.01] nor an interaction [*F*(2,52) = 1.64, *p* = 0.210, ε = 0.80, η_p_^2^ = 0.06; baby: pre *M* ± *SEM* = 71.69 ± 3.36 s, post *M* ± *SEM* = 76.16 ± 5.54 s, pressure *M* ± *SEM* = 71.41 ± 4.11 s; adult: pre *M* ± *SEM* = 83.12 ± 5.53 s, post *M* ± *SEM* = 78.62 ± 8.28 s, pressure *M* ± *SEM* = 84.05 ± 8.89 s].

As a visual inspection suggested that the adult-animal pictures group showed longer operation time than the baby-animal pictures group in the pre-test (Figure 2), we analyzed operation times in both the post-test and the pressure test, using an ANCOVA with mean operation time in the pre-test as a covariate. A two-way ANCOVA did not reveal group difference [*F*(1,25) = 0.34, *p* = 0.57, η_p_^2^ = 0.01] or a main effect of test [*F*(1,25) = 0.17, *p* = 0.681, η_p_^2^ = 0.01]. However, a significant interaction between the group and test was noted [*F*(1,25) = 6.54, *p* = 0.017, η_p_^2^ = 0.21]. Bonferroni-adjusted *post hoc* tests for the baby-animal pictures group showed a marginally longer time in the post-test than in the pressure test (*p* = 0.083), whereas the adult-animal pictures group showed a marginally longer time in the pressure test than in the post-test (*p* = 0.067). There was no difference in operation time between the groups in either test (post-test: *p* = 0.224; pressure test: *p* = 0.882).

### State Anxiety

State anxiety scores were subjected to a two-factor mixed design ANOVA with group (baby/adult animals) as a between-subjects factor and test (pre/post/pressure) as a within-subjects factor (Table 2).

**TABLE 2:** Mean scores of state anxiety before each test (standard error of mean in parentheses).

	Pre	Post	Pressure
Baby	41.73 (2.70)	54.53 (2.51)	52.13 (2.44)
Adult	39.54 (2.14)	51.0 (2.54)	51.54 (2.17)

The main effect of test was significant [*F*(2,52) = 13.13, *p* < 0.001, ε = 0.70, η_p_^2^ = 0.34]. *Post hoc* tests revealed higher state anxiety in both the post-test (*M* ± *SEM* = 52.89 ± 1.79) and the pressure test (*M* ± *SEM* = 51.86 ± 1.62), than in the pre-test (*M* ± *SEM* = 40.71 ± 1.73; *p*s < 0.01). There were no differences between groups [*F*(1,26) = 1.58, *p* = 0.221, η_p_^2^ = 0.06], and no interaction was found [*F*(2,52) = 0.16, *p* = 0.778, ε = 0.70, η_p_^2^ = 0.01].

### QE Duration

**FIGURE 3:**
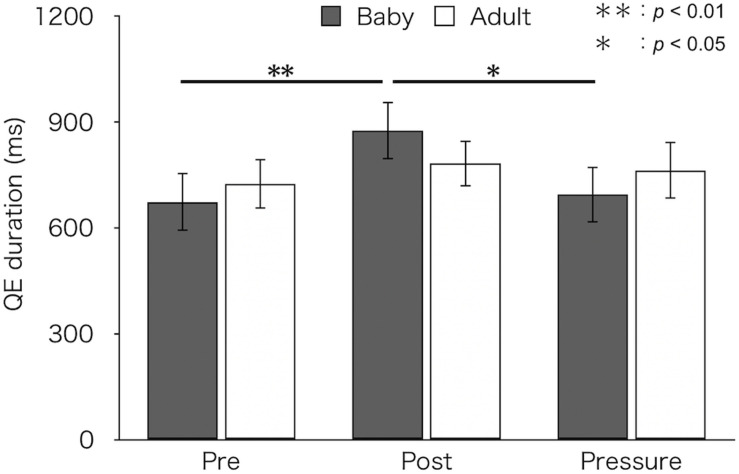
Mean QE duration of the two groups (Baby vs. Adult) in each test. Error bars indicate standard error of the mean (SEM).

Figure 3 depicts mean QE durations for each test and group. A two-factor mixed design ANOVA found a main effect of test [*F*(2,52) = 6.18, *p* = 0.004, η_p_^2^ = 0.19]. There was no difference in QE duration between groups [*F*(1,26) = 0.01, *p* = 0.93, η_p_^2^ < 0.01]. An interaction between group and test was marginally significant [*F*(2,52) = 2.66, *p* = 0.079, η_p_^2^ = 0.09]. A simple main effect of test was found in the baby-animal pictures group [*F*(2,52) = 8.92, *p* < 0.001], but not in the adult-animal pictures group [*F*(2,52) = 0.52, *p* = 0.596]. Bonferroni-adjusted *post hoc* tests showed significantly longer QE duration in the post-test (*M* ± *SEM* = 876 ± 82 ms) than both in the pre-test (*M* ± *SEM* = 674 ± 83 ms) and the pressure test (*M* ± *SEM* = 694 ± 79 ms) for the baby-animal pictures group (*p* < 0.01 and *p* = 0.014, respectively).

**FIGURE 4:**
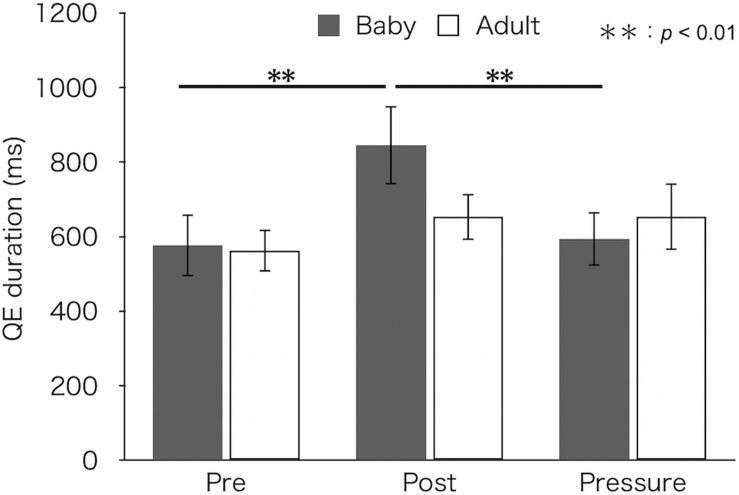
Median QE duration of the two groups (Baby vs. Adult) in each test. Error bars indicate standard error of the mean (SEM).

Figure 4 depicts median QE durations for each test and group. A two-factor mixed design ANOVA found a main effect of test [*F*(2,52) = 7.27, *p* = 0.002, η_p_^2^ = 0.22]. There was no difference in QE duration between groups [*F*(1,26) = 0.23, *p* = 0.634, η_p_^2^ = 0.01]. Furthermore, the interaction between group and test was significant [*F*(2,52) = 3.61, *p* = 0.034, η_p_^2^ = 0.12]. A simple main effect of test was found in the baby-animal pictures group [*F*(2,52) = 10.45, *p* < 0.001], but not in the adult-animal pictures group [*F*(2,52) = 1.10, *p* = 0.341]. Bonferroni-adjusted *post hoc* tests for the baby-animal pictures group showed a significantly longer QE duration in the post-test (*M* ± *SEM* = 846 ± 106 ms) than in both the pre- (*M* ± *SEM* = 577 ± 83 ms) and pressure (*M* ± *SEM* = 594 ± 73 ms) tests (*p*s < 0.01).

**FIGURE 5:**
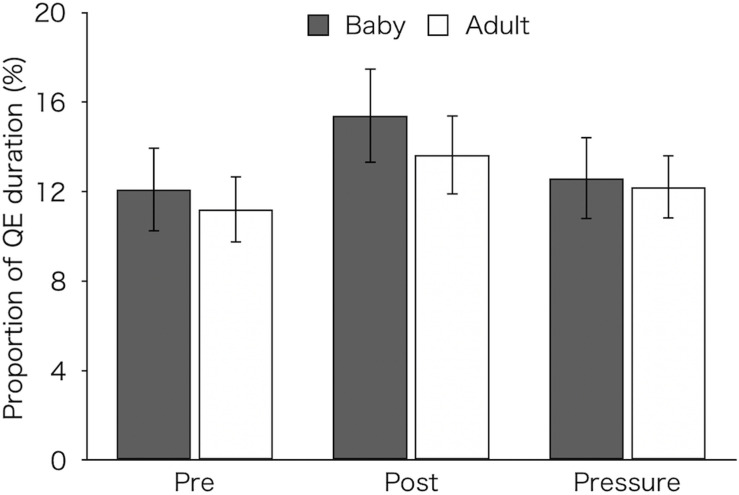
Proportion of QE duration of the two groups (Baby vs. Adult) that were calculated by dividing the total QE durations over 12 pick-ups by the total operation time in each test. Error bars indicate standard error of the mean (SEM).

Figure 5 shows the proportion of QE duration that were calculated by dividing the total QE durations over 12 pick-ups by the total operation time in each test. A two-factor mixed design ANOVA revealed a main effect of test [*F*(2,52) = 7.37, *p* = 0.002, η_p_^2^ = 0.22]. *Post hoc* tests showed that the proportion of QE (*M* ± *SEM* = 14.58 ± 1.34%) was significantly higher in the post-test than in both the pre-test (*M* ± *SEM* = 11.67 ± 1.17%) and pressure test (*M* ± *SEM* = 12.42 ± 1.13%) (*p* < 0.01 and *p* = 0.026, respectively). Neither a main effect of group [*F*(1,26) = 0.18, *p* = 0.676, η_p_^2^ = 0.01] nor an interaction [*F*(2,52) = 0.4, *p* = 0.672, η_p_^2^ = 0.02] was found.

**FIGURE 6:**
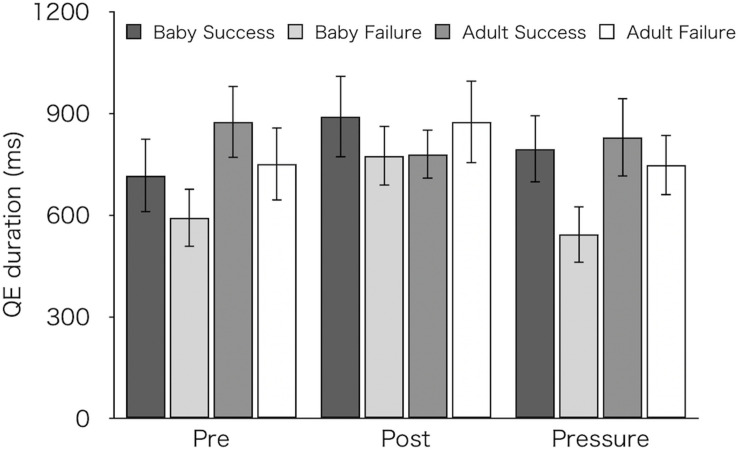
Mean QE durations for successful and failed pick-ups of the two groups (Baby vs. Adult) in each test. Error bars indicate standard error of the mean (SEM).

Figure 6 shows mean QE durations for succeeded and failed pick-ups. We compared QE durations between succeeded and failed pick-ups. For this analysis, we excluded one participant from each group (resulting in 14 for the Baby group and 12 for the Adult group) who did not achieve any success in the pre-test. A three-factor mixed design ANOVA revealed significantly longer QE durations on succeeded pick-ups (*M* ± *SEM* = 815 ± 61 ms) than on failed pick-ups (*M* ± *SEM* = 714 ± 55 ms) [*F*(1,24) = 5.63, *p* = 0.026, η_p_^2^ = 0.19]. There were no main effects of test [*F*(2,48) = 2.18, *p* = 0.124, η_p_^2^ = 0.08] and of group [*F*(1,24) = 0.70, *p* = 0.41, η_p_^2^ = 0.03], and no interactions [test × group: *F*(2,48) = 1.24, *p* = 0.299, η_p_^2^ = 0.05; success/failure × group: *F*(1,24) = 2.25, *p* = 0.147, η_p_^2^ = 0.09; test × success/failure: *F*(2,48) = 1.33, *p* = 0.273, η_p_^2^ = 0.05; three-way interaction: *F*(2,48) = 0.64, *p* = 0.532, η_p_^2^ = 0.03].

## Discussion

We examined whether viewing cute pictures could influence QE duration as well as performance in both a post-test and pressure test. We obtained similar findings to Sherman et al. (2009) and Nittono et al. (2012), indicating that participants’ performance in the task significantly increased after viewing cute pictures. However, performance did not change after viewing pictures of adult animals. We also found a longer QE duration after viewing baby-animal pictures in the post-test. Regarding pressure manipulation, we failed to induce a significantly higher state anxiety in the pressure test than in the post-test. Therefore, we cannot decide whether viewing cute pictures mitigates performance deterioration by choking under pressure. Nevertheless, performance improvement was also observed in the pressure test in the baby-animal pictures group, whereas the QE lengthening effect disappeared under pressure. In general, QE durations were longer for successful pick-ups than for failed pick-ups, which was in accordance with previous studies (Vickers, 1992, 1996; Wilson and Pearcy, 2009), suggesting the coexistence of transient changes of QE duration in a state raised by viewing pictures.

According to previous studies (Sherman et al., 2009; Nittono et al., 2012), we selected pictures that differed in subjective rating scores on cuteness, infantility, and wanting to get closer between the baby- and adult-animal pictures groups. To reconfirm the validity of picture selection, we analyzed subjective ratings on these items after the experiment. As expected, we found significantly higher scores for the baby-animal pictures group than for the adult-animal pictures group, reconfirming the validity of our picture selection.

Given our success in replicating previous findings on controlling emotions (Sherman et al., 2009; Nittono et al., 2012), it could be concluded that viewing cute pictures improves the performance of fine motor skills, as demonstrated by finger pinching in our study. Using a global–local letter task, Nittono et al. (2012) demonstrated that participants learned to focus more on the local aspect than on the global aspect of the letter stimulus after viewing baby-animal pictures. Thus, focus of external attention is thought to be responsible for the beneficial effect of viewing cute pictures. In the context of attention control, previous studies also asserted that perceiving cuteness may increase perceptual carefulness (Nittono et al., 2012) and tenderness (Sherman et al., 2009) in motor behavior that requires dexterity. It should be noted that the beneficial effects on motor performance might be confined to fine skills, because grasping force in the hands (i.e., more ballistic and gross motor tasks) was not shown to be influenced by viewing cute pictures (Sherman et al., 2009). Additionally, the beneficial effects appear to be independent of arousal level, as neither skin conductance level nor heart rate changed while viewing cute pictures (Sherman et al., 2009).

Although state anxiety scores were higher in both the post-test and pressure test than in the pre-test (i.e., baseline), better performance associated with viewing cute pictures was observed, even in the high-pressure situation. However, enhanced state anxiety did not differ between the post-test and pressure-test regardless of group, suggesting either a null effect of our pressure manipulation or a ceiling effect of anxiety in the pressure test. According to introspective reports, some participants claimed that the task was more difficult than expected when they performed it in the pre-test, and, thus, the state anxiety might have been abruptly increased in the post-test. Additionally, participants might not perceptibly dissociate the difficulties of the task among tests because the trial (i.e., 12 pick-ups) was completed in a short period of time (75–90 s). Nevertheless, it should be emphasized that the beneficial effects of viewing cute pictures could be obtained even in a situation where performance was being evaluated.

In a previous study, the effect of viewing cute pictures was associated with an extension of time to complete the task as well as performance (Nittono et al., 2012). Thus, it is worth considering the involvement of the speed–accuracy trade-off in our results. Although we found significant differences in score rate between the two groups, both in the post- and pressure tests, we did not find any difference in the operation time between the groups (even while considering operation time in the pre-test as a covariate). If speed–accuracy trade-off had occurred, a significantly longer operation time would have been observed in the baby-animal pictures group. This was not the case in our study. Therefore, our results may not have been caused by a speed–accuracy trade-off.

Importantly, QE duration became longer after viewing cute pictures in the post-test, compared with the pre-test. This supported our hypothesis that viewing cute pictures facilitates top-down attention control and should improve performance and prolong QE duration. Longer QE duration was not observed after viewing pictures of adult animals. Considering both the improvement and group differences in performance, it is plausible that viewing cute pictures strengthened top-down attention control in both the post-test and pressure test. Longer QE duration in the post-test might have been facilitated by viewing cute pictures, given that attention control is responsible for prolongation of QE duration (Vine and Wilson, 2010, 2011; Vine et al., 2011). However, a previous study reported that instructions inducing external attention improved performance but did not influence QE durations (Klostermann et al., 2014), suggesting that external attention may not be a critical factor for prolongation of QE. Gonzalez et al. (2017b) assert that a possible underlying mechanism of the beneficial effect of QE that relies on attentional allocation has not been empirically ascertained, although external attention might affect QE durations for some aspects. We did not give participants explicit instructions that could induce focus of external attention. Thus, it is possible that longer QE durations in the post-test were not due to external attention. Instead, top-down attention control induced by viewing cute pictures might have affected both performance and QE durations in the post-test.

However, longer QE durations were not observed in the pressure test, although preciseness was higher in the pressure test than in the pre-test. The discrepancy between performance and QE durations might have also been due to instructions used in our study. We gave participants instructions that emphasized on manipulative aspects of the fine motor task but did not include any attention-related aspects. Thus, the participants were not explicitly oriented to the objects to be picked up by the instruction, resulting in improvement of performance. In QE training, instructions about gaze behaviors are given to participants, and thus focus of attention might be enhanced explicitly by the instructions (Gonzalez et al., 2017b). This may result in prolongation of QE durations. In contrast, the participants in our study did not prolong QE durations in the pressure test without such instructions. Although the influence of our pressure manipulation was not clear, it might have resulted in the retrograding QE in the pressure test. Furthermore, most previous studies have adopted relatively long QE training periods (e.g., more than 3 days) to prolong QE duration in a severe pressure situation (Vine and Wilson, 2010, 2011). Thus, adequate time may be needed to acquire proper QE, shifting from conscious to autonomous gaze behaviors. Although state anxiety did not differ between the post- and pressure tests, it is possible that evaluation by the experimenter might have interrupted prolongation of QE duration in the pressure test. Previous studies have reported shorter QEs in various pressure conditions (Behan and Wilson, 2008; Wilson et al., 2009). The current results suggested that viewing cute pictures is not enough to prolong QE duration in a high-pressure situation. Therefore, instruction-based training lasting several days should be adopted to properly acquire QE.

If participants implicitly perceived more pressure in the pressure test than in the post-test that was not reflected in state anxiety scores, shortened QE durations after viewing cute pictures in the pressure test might have represented the vulnerability of QE to pressure-inducing procedures, whereas the kawaii-viewing effect on performance may not be influenced by pressure manipulation. Therefore, another possible explanation for our results may be that QE and the effects of viewing cute pictures do not represent the same phenomenon, although both are associated with attention control. Maintaining longer QE while playing sports is an effective strategy to prevent performance deterioration. However, viewing cute pictures may increase approach motivation as well as attention to the current task, yielding performance improvement. QE is more associated with preventing performance deterioration, whereas the effects of cuteness are more associated with performance improvement (e.g., so-called “clutch” phenomenon, Otten, 2009). This might help to explain why we found a discrepancy between QE durations and performance in the pressure test.

Thus, our findings provide an important implication that viewing cute pictures may have an immediate effect on performance improvement, even under pressure. As was observed in the post-test, viewing cute pictures seemed to prolong QE duration as well as improve performance, in line with earlier studies that found a positive correlation between longer QEs and better performance (Vine et al., 2013). The finding that performance improvement remained even though QE duration became shorter in the pressure test suggested that viewing cute pictures might be a more efficient and effective way to prevent choking under pressure than QE training. Further studies are needed to confirm the relationship between QE and effects of viewing cute pictures and examine if viewing cute pictures can improve performance in gross motor tasks (i.e., actual sports skills).

Finally, we summarize the limitations of our study. Since our pressure manipulation did not function adequately, more effective ways should be considered in the future studies to evaluate the utility of viewing cute pictures. Additionally, our results did not explicitly reveal that simply viewing cute pictures can prolong QE durations via attention control, even in a pressure situation. Thus, further research should shed light on the relationship between performance improvement caused by viewing cute pictures and QE duration, in relation to pressure.

## Data Availability Statement

The datasets generated for this study are available on request to the corresponding author.

## Ethics Statement

This study was carried out in accordance with the recommendations of the Waseda University Ethics Committee with written informed consent from all participants. The protocol was approved by the Waseda University Ethics Committee.

## Author Contributions

NY and HM contributed to the conception and design of the study. NY collected and analyzed the data. HM, HN, and NY drafted the manuscript. All authors approved the version of the manuscript to be published.

## Conflict of Interest

The authors declare that the research was conducted in the absence of any commercial or financial relationships that could be construed as a potential conflict of interest.
